# Nurse Researchers; an Insight in Science Production in Ophthalmology

**Published:** 2013

**Authors:** Bajic Vladan

**Affiliations:** University of Belgrade, Belgrade, Serbia

**Keywords:** Nurse Researcher, Ophthalmology and visual sciences, Science production

Great advancements in ophthalmology and visual sciences have been occurred in recent decades through advantages of globalization. There has been a great evolution in ocular health however, there are still many problems associated with ophthalmic health around the world. World Health Organization (WHO) in collaboration with the International Agency for Preventing Blindness (IAPB) launched an initiative to prevent etiologies of blindness by the year 2020 which has been titled as VISION 2020: the Right to Sight. The program targets blindness and its credo is that sight should be recognized as a fundamental human right. It is imperative that the various institutes and agencies in commissioning and launching new aims, strategies and monitoring programs for success in the right to sight collaborate in order to best address the many challenges that the ophthalmic discipline is presently dealing with Ideally, VISION 2020 is made a compulsory activity for all institutions in the ophthalmic health community [1-3].

Ophthalmology as one of the most critical specialties in medical science should be given due consideration in any health care system. It is a globally accepted fact that the smallest mistakes in treating patients in this discipline could cause tragic and severe consequences such as loss or impairment of vision. It is important that the ophthalmic nurses' work along with ophthalmologists to ensure the necessary level of care and programs need to be developed to improve their knowledge base in handling patients. A well trained ophthalmic nurse working as a practitioner would significantly lessen the burden of care on the health care system in areas that have no immediate access to ophthalmologists. At a minimum, those nurses could be of tremendous value if they were taught and could act in the role of differentiating between emergency and non-emergency visual care. The nurse could have a critical role in making the appropriate referrals for patients who must be seen by ophthalmic specialists in emergency cases. Such a strategy would save crucial time (in emergency situations), serve to better deploy and allocate services as well as reducing the overall financial expense of ophthalmic care.

Improving public awareness through dissemination of information is crucial in establishing a healthy society and now is a common approach. Nurses in the health care system have the most frequent personal contact with patients and are ubiquitous throughout medical centers, clinics, and hospitals. Programs that will improve nurses' knowledge will in turn help them to teach people about important ophthalmic health matters. Due to the insufficient time available to most physicians, there is need for well-informed individual in dealing and handling clients. Most of this gap could be addressed by having trained ophthalmic nurses work with patients who have ocular problems in the medical centers. 

The International Council of Nurses was founded in 2002 to address the need for qualified care and to develop a basis and template that would allow nurses to share their information, experiences and knowledge, while globally encouraging them to improve their abilities through communications. This foundation has made a great contribution in promoting teamwork among nurses and acts as stimuli to encourage them in multi-center research while helping each other in the health and medical area [4-5]. Currently, evidence based medicine makes the decision of choosing one or another medical treatment. On that basis, there are many health and medical centers that seek programmed research and studies in order to establish a well-documented system. In this respect, nurse researchers could fill the gap between practice and research [6-8]. They could work on controversial issues as it relates to health and disease to give priority to those topics in research. Moreover, these trained nurses may play a critical role in data collection to further promote the science production process. As assistants to researchers, in light of their experience in the handling of and personal contact with patients, ophthalmic nurses can see problems in healthcare systems much better than others as they are in the practical world and are aware of the inefficiencies and shortcomings in the system [6]. Therefore, by improving the practical skills of nurses with the research abilities, this group of healthcare workers would be a great asset to physicians and medical world. Nurses are able to improve patients' information on the medical issues, teach them about risks and discuss the factors, symptoms and signs of diseases that could help patient to understand the nature of his problem and prevent unnecessary poor consequences. The public typically pays more attention to interventions and knowledge sharing if these efforts are founded on documented real life evidence. Better public health could be achieved if information is disseminated in that manner.

Another helpful program is using nurses' abilities to arrange collaborative sessions and workshops in which they could discuss and share their knowledge with people in various areas of health care. By using the most common and critical health topics, those workshops could address many present medical problems such as diabetes, venereal diseases, obesity, diabetes, and hypertension, which would be of great interest to those affected and in those fields of medicine. Also, nurse researchers may have a potential role in handling special workshops for physicians regarding novel issues of health and general management. Therefore they can help physicians to further educate themselves and in turn, the nurses themselves will be able to become familiar with the most up to date findings in medical subjects related to their fields of activities.

Consequently, one could easily see that in present medical system, the nurse researchers would be a significant contribution towards improving the health system and making the nursing society directly engaged in research will be an extraordinary improvement in manpower as well as overall scientific production.

In the autumn issue of Medical Hypothesis, Discovery & Innovation Ophthalmology Journal, you will have a chance to study the relationship between Aspirin and AMD, a review of en-face choroidal imaging using spectral-domain OCT, a modified color vision test, the genetics of AMD, and ocular higher order aberrations in ptotic children. Additionally, the coming winter issue has many good articles developed by prestigious international researchers in various fields. 

A fair and justified process of reviewing papers, adopting best research made on newly introduced titles and the diversity in writing papers as well as more focus on controversial issues had been integrated to journal policy. 

As a final point, we look forward to your valuable opinions so that we can continue to improve the Journal.

## DISCLOSURE

Conflicts of Interest: None declared.
